# Relationships within Cladobranchia (Gastropoda: Nudibranchia) based on RNA-Seq data: an initial investigation

**DOI:** 10.1098/rsos.150196

**Published:** 2015-09-23

**Authors:** Jessica A. Goodheart, Adam L. Bazinet, Allen G. Collins, Michael P. Cummings

**Affiliations:** 1 Laboratory of Molecular Evolution, Center for Bioinformatics and Computational Biology, University of Maryland, College Park, MD 20742, USA; 2 NMFS, National Systematics Laboratory, National Museum of Natural History, Smithsonian Institution, MRC-153, PO Box 37012, Washington, DC 20013, USA

**Keywords:** Mollusca, phylogenomics, nudibranchs, sea slugs, RNA-Seq, phylotranscriptomics

## Abstract

Cladobranchia (Gastropoda: Nudibranchia) is a diverse (approx. 1000 species) but understudied group of sea slug molluscs. In order to fully comprehend the diversity of nudibranchs and the evolution of character traits within Cladobranchia, a solid understanding of evolutionary relationships is necessary. To date, only two direct attempts have been made to understand the evolutionary relationships within Cladobranchia, neither of which resulted in well-supported phylogenetic hypotheses. In addition to these studies, several others have addressed some of the relationships within this clade while investigating the evolutionary history of more inclusive groups (Nudibranchia and Euthyneura). However, all of the resulting phylogenetic hypotheses contain conflicting topologies within Cladobranchia. In this study, we address some of these long-standing issues regarding the evolutionary history of Cladobranchia using RNA-Seq data (transcriptomes). We sequenced 16 transcriptomes and combined these with four transcriptomes from the NCBI Sequence Read Archive. Transcript assembly using Trinity and orthology determination using HaMStR yielded 839 orthologous groups for analysis. These data provide a well-supported and almost fully resolved phylogenetic hypothesis for Cladobranchia. Our results support the monophyly of Cladobranchia and the sub-clade Aeolidida, but reject the monophyly of Dendronotida.

## 1. Introduction

Cladobranchia is a diverse (approx. 1000 species) but understudied and poorly understood group of sea slug molluscs. It is also a clade within Nudibranchia, which is a group of marine gastropods defined by the loss of the adult shell [1]. In the absence of a protective shell, these slug lineages have evolved a diverse array of alternative defence mechanisms. The development of chemical and physical defence mechanisms has allowed for the occupation of new ecological niches [2] and has been hypothesized as having been a primary driver in the diversification of Nudibranchia [1]. Defensive strategies found in Cladobranchia include the uptake or synthesis of biochemically active compounds [3,4], the presence of warning (aposematic) coloration [5] or cryptic coloration to deter or hide from predators, respectively, and the use of stinging organelles (nematocysts) acquired from cnidarian prey [1,6]. The theft and synthesis of biochemically active compounds has provided a pool of materials that have potential uses in the synthesis of new pharmaceuticals (e.g. Zalypsis, currently in phase II clinical trials for the treatment of various cancers; made from a chemical isolated from *Jorunna funebris*) [7–9]. Strong phylogenetic hypotheses of Cladobranchia will be useful in understanding the evolution of these chemical defences and the evolution of other character traits, such as the ability of many cladobranch species to sequester nematocysts [6] and swimming behaviours [10].

To date, there have been only two large-scale phylogenies published specifically on Cladobranchia [11,12], the first of which used the three genes most commonly used in nudibranch phylogenetics (mitochondrial 16S rRNA and cytochrome oxidase I (COI), and nuclear histone H3) [11]. The second study used all data publicly available through GenBank [13] to understand the evolutionary relationships of members of this group, thus adding two additional genes and increasing taxon sampling by approximately 200 taxa [12]. In both of these phylogenetic inferences of Cladobranchia, the majority of relationships remained unclear, both between and within the three traditional taxonomic divisions of Cladobranchia (Arminida, approx. 100 species; Dendronotida, approx. 250 species; and Aeolidida, approx. 600 species). The name of the taxon Arminida was changed by Bouchet & Rocroi [14], who introduced the taxon Euarminida for the two families Arminidae and Doridomorphidae. Though the analysis by Pola & Gosliner [11] supported the inclusion of only those families within a clade, they disagreed with the name change and retained Arminida in an altered sense, which we follow.

In addition to the phylogenies specifically aimed at understanding relationships within Cladobranchia, phylogenetic inferences have been attempted to address the evolutionary history of Nudibranchia or the larger clade Euthyneura [15–21]. The results regarding the major groupings within Cladobranchia were inconsistent in all of these studies, with all three major divisions considered both paraphyletic and monophyletic in different publications. The most recent and comprehensive work by Wollscheid-Lengling *et al*. [16], based on the 18S, 16S and COI genes, suggested that Aeolidida is monophyletic and both Dendronotida and Arminida are paraphyletic.

Though the more basal evolutionary history of Cladobranchia has been problematic for phylogenetic inference, a number of studies (both morphological and molecular) have provided evidence to support relationships at both the family and generic levels. Phylogenies of individual families have been published on Aeolidiidae [22], Arminidae [23,24], Bornellidae [25], Glaucidae [26], Scyllaeidae [27] and Tritoniidae [28], and at the genus level publications have focused on *Antaeolidiella* [29], *Babakina* [30,31], *Berghia* [32], *Burnaia* [33], *Dendronotus* [34–36], *Limenandra* [37], *Melibe* [38], *Phyllodesmium* [39,40] and *Spurilla* [41]. All of these studies were focused on particular aspects of the Cladobranchia tree, and although they have supported some relationships, it is clear that the overall relationships of higher level groups in Cladobranchia are not well understood. In addition, individually these studies cover very little of the overall diversity of Cladobranchia.

Most importantly for the purposes of this paper, the phylogenies estimated in all previous studies on taxa within Cladobranchia have lacked the support needed for confidence in suprageneric taxonomic relationships, with the exception of a small subset of familial-level phylogenies, mentioned above. This lack of resolution and low overall bootstrap (BS) support demonstrates that the relatively small, multi-gene strategies that have been previously used for phylogenetic reconstruction are insufficient for comprehensive analyses and deep phylogenetic inferences regarding the relationships of this particular group. To address this, our paper presents a preliminary exploration into the use of RNA-Seq data to generate well-supported hypotheses regarding the evolutionary relationships within Cladobranchia. This study includes the publication of 16 new cladobranchian transcriptomes.

## 2. Material and methods

### 2.1 Organismal sampling

**Figure 1.**
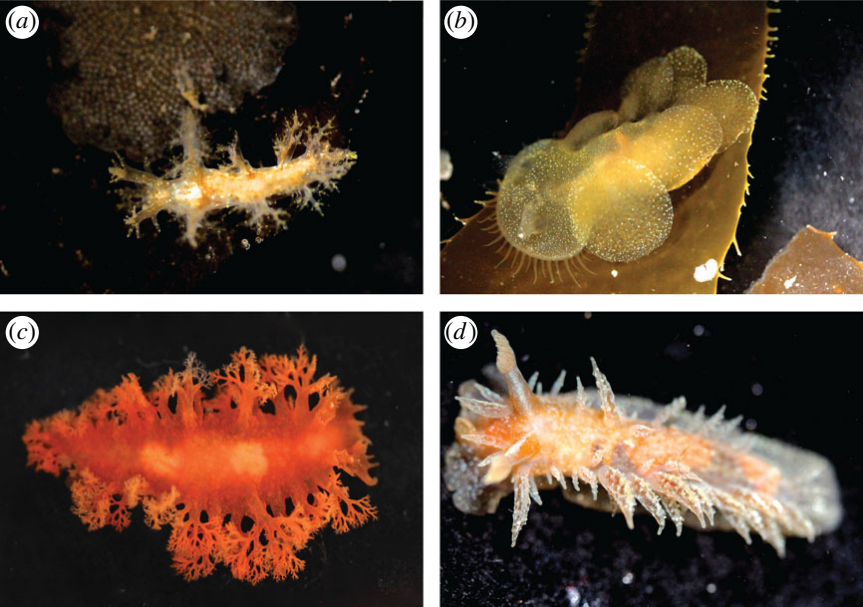
Select photographs of dendronotid and unassigned taxa used in this project, including: (*a*) *Dendronotus venustus* (SRR1950948), (*b*) *Melibe leonina*, (*c*) *Tritoniopsis frydis* (SRR1950954) and (*d*) *Dirona picta* (USNM1276030).

**Figure 2.**
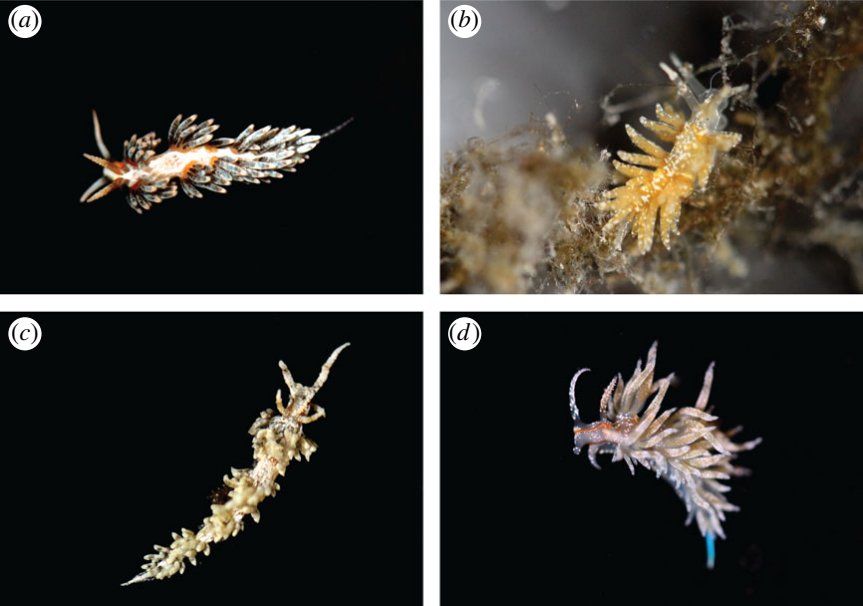
Select photographs of aeolid taxa used in this project, including: (*a*) *Berghia stephanieae* (SRR1950951), (*b*) *Favorinus auritulus* (USNM1276034), (*c*) *Palisa papillata* (SRR1950952) and (*d*) *Dondice occidentalis*.

Two specimens of each representative species (a total of 16) were collected in tide pools or via snorkelling or SCUBA (under AAUS certification) using a variety of methods (direct collection, substrate collection and non-destructive collecting under rocks), with one individual used for RNA-Seq and one individual preserved as a voucher and deposited in the Smithsonian National Museum of Natural History (SI-NMNH). Some specimen photographs are shown in figures 1 and 2. We generated raw transcriptome data by RNA-Seq for 16 Cladobranchia species and downloaded data for one additional Cladobranchia species from the US National Center for Biotechnology Information (NCBI) Sequence Read Archive (SRA). Three outgroup transcriptomes were also obtained from the SRA: two representatives of Anthobranchia (the sister taxon of Cladobranchia) and one of Pleurobranchoidea (the sister taxon to Nudibranchia). Specimen and sequence data are listed in table 1.

**Table 1.** List of specimens examined in this study, including species name, locality and morphological voucher information. SRA accession numbers are also provided for each transcriptome.

species	locality	morphological voucher	SRA accession no.
*Bathydoris clavigera*		NCBI SRA	SRR1505104
*Doris kerguelenensis*		NCBI SRA	SRR1505108
*Fiona pinnata*		NCBI SRA	SRR1505109
*Pleurobranchaea californica*		NCBI SRA	SRR1505130
*Austraeolis stearnsi*	Point Loma, San Diego, CA, USA	USNM1276025	SRR1950943
*Berghia stephanieae*	Key Largo, FL, USA	—	SRR1950951
*Catriona columbiana*	Redondo Beach, CA, USA	—	SRR1950949
*Cuthona albocrusta*	Mission Bay, San Diego, CA, USA	USNM1276026	SRR1950944
*Dendronotus venustus*	Morro Bay, CA, USA	USNM1276033	SRR1950948
*Dirona picta*	Redondo Beach, CA, USA	USNM1276030	SRR1950946
*Dondice occidentalis*	Riviera Beach, FL, USA	USNM1276036	SRR1950953
*Doto lancei*	Mission Bay, San Diego, CA, USA	USNM1276027	SRR1950945
*Favorinus auritulus*	Key Largo, FL, USA	USNM1276034	SRR1950950
*Flabellina iodinea*	Point Loma, San Diego, CA, USA	USNM1276023	SRR1950940
*Hermissenda crassicornis*	Sunset Cliffs, San Diego, CA, USA	USNM1276022	SRR1950939
*Janolus barbarensis*	Point Loma, San Diego, CA, USA	USNM1276029	SRR1950942
*Melibe leonina*	Morro Bay, CA, USA	USNM1276031	SRR1950947
*Palisa papillata*	Key Largo, FL, USA	—	SRR1950952
*Tritonia festiva*	Point Loma, San Diego, CA, USA	USNM1276024	SRR1950941
*Tritoniopsis frydis*	Pompano Beach, FL, USA	USNM1276038	SRR1950954

A visual examination was used for confirmation of identity using field guides published by Valdés *et al*. [42] (for the Caribbean) and Behrens & Hermosillo [43] (for the eastern Pacific), as well as expert opinions when the placement of species was uncertain. One of the two specimens was placed in RNAlater solution (Qiagen, Hilden, Germany) for RNA preservation and frozen within one week of collection at −80°C to prevent RNA degradation. Some specimens in RNAlater were refrigerated or placed at −20°C within 24 h, others were kept at room temperature for up to one week. A second specimen of each species was preserved as a voucher for morphological analysis, first in Bouin's Fixative and subsequently transferred to 70% ethanol for long-term storage. Voucher specimens were deposited in the SI-NMNH and are available for study under the catalogue numbers provided in table 1.

### 2.2 RNA extraction and sequencing

A 20–100 mg tissue sample was taken from the anterior of each animal and manually homogenized using a motorized pestle. After 1–2 min of homogenizing, the tissue was flash frozen in liquid nitrogen for subsequent homogenizing, until tissue mixture was fully uniform. Five hundred microlitres of TRIzol Reagent (Life Technologies, Carlsbad, CA, USA) was then added and the mixture was homogenized again. This procedure was repeated until the solution was fully homogenized. Once this process was complete, an additional 500 μl of TRIzol Reagent was added to the solution and the mixture was left at room temperature for 5 min.

One hundred microlitres of bromochloropropane was then added to the solution, which was subsequently mixed thoroughly. The mixture was left at room temperature for 5 min, then centrifuged at 16 000*g* for 20 min at 8°C. Following this step, the top aqueous phase was removed and placed in another tube where 500 μl of 100% isopropanol was added. This tube was stored overnight at −20°C for RNA precipitation.

After overnight precipitation, the samples were centrifuged at 17 200*g* for 10 min at 4°C. The supernatant was then removed and the pellet washed with freshly prepared 75% ethanol. The sample was then centrifuged at 7500*g* for 5 min at 4°C. The supernatant was removed and the pellet air-dried for 1–2 min (or until it looked slightly gelatinous and translucent). The total RNA was then re-suspended in 10–30 μl of Ambion Storage Solution (Life Technologies) and 1 μl of RNase inhibitor (Life Technologies) was added to prevent degradation.

Total RNA samples were submitted to the DNA Sequencing Facility at University of Maryland Institute for Bioscience and Biotechnology Research, where quality assessment, library preparation and sequencing were completed. RNA quality assessment was done with a Bioanalyzer 2100 (Agilent Technologies, Santa Clara, CA, USA), and total RNA samples with a concentration higher than 50 ng μ*l*^−1^ were used for library construction. For library preparation, the facility used the Illumina TruSeq RNA Library Preparation Kit v2 (Illumina, San Diego, CA, USA) and 200 bp inserts; 100 bp, paired-end reads were sequenced with an Illumina HiSeq1000 (Illumina).

### 2.3 Quality control and assembly of reads

Reads that failed to pass the Illumina ‘Chastity’ quality filter were excluded from our analyses. Subsequent quality assessment and control were performed using autoadapt (v. 0.2 [44]) with default settings, which in turn used FastQC (v. 0.10.1 [45]) and cutadapt (v. 1.3 [46]) to remove overrepresented sequences and to trim and remove low-quality reads. Reads passing quality control were assembled using Trinity (v. r20140717 [47]) with default settings, which required assembled contigs to be at least 200 bp long.

### 2.4 Orthology assignment

Translated transcript fragments were organized into orthologous groups corresponding to a custom gastropod-specific core-orthologue set (3854 protein models) using HaMStR (v. 13.2.2 [48]), which in turn used FASTA (v. 36.3.6d [49]), GeneWise (v. 2.2.0 [50]) and HMMER (v. 3.0 [51]). In the first step of the HaMStR procedure, substrings of assembled transcripts (translated nucleotide sequences) that matched one of the gastropod protein models were provisionally assigned to that orthologous group. To reduce the number of highly divergent, potentially paralogous sequences returned by this search, we set the *E*-value cut-off defining a hidden Markov model (HMM) hit to 1×10^−5^ (the HaMStR default is 1.0) and retained only the top-scoring quartile of hits. In the second HaMStR step, the provisional hits from the HMM search were compared to our reference taxon, *Aplysia californica*, and retained only if they survived a reciprocal best BLAST [52] hit test with the reference taxon using an *E*-value cut-off of 1×10^−5^ (the HaMStR default was 10.0). In our implementation, we substituted FASTA [49] for BLAST because FASTA programs readily accepted our custom substitution matrix (GASTRO50).

The gastropod core-orthologue set was generated by first downloading all available gastropod clusters with 50% similarity or higher from UniProt [53] (39 403 clusters). Excluding clusters that contained only one sequence left 6160 clusters. We calculated the sequence similarity of each cluster and, as a heuristic, decided to remove clusters whose percentage identity was less than 70%, which left 6015 clusters. We then assessed the number of times each taxon was represented within those clusters. *Aplysia californica* was identified as the most abundant taxon (3854 associated clusters with 70% similarity or higher) and was therefore selected as the reference taxon for the custom HaMStR database. We constructed the gastropod HaMStR database by following the steps given in the HaMStR README file, which included generating profile HMMs for each cluster using HMMER. Our gastropod HaMStR database contained 3854 orthologous groups. All protein sequences for *A. californica* (Uniprot/NCBI taxon ID 6500) were downloaded from UniProt and used to generate the BLAST database for HaMStR.

Construction of the custom substitution matrix (GASTRO50) followed the procedure outlined in Lemaitre *et al*. [54] and used the 50%-similarity gastropod clusters downloaded from UniProt. In this protocol, a block is defined as a conserved, gap-free region of the alignment. Our blocks output file contained 34 109 blocks and a total of 2 442 130 amino acid positions. (This was after we removed one large block from the blocks output file that contained 1388 sequences, which prevented the scripts from executing properly.)

### 2.5 Construction of data matrix and paralogy filtering

Protein sequences in each orthologous group were aligned using MAFFT (v. 7.187 [55]). We used the ---auto and ---addfragments options of MAFFT to add transcript fragments to the *A. californica* reference sequence, which was considered the existing alignment. We converted the protein alignments to corresponding nucleotide alignments using a custom Perl script. A maximum-likelihood tree was inferred for each orthologous group using GARLI (v. 2.1 [56]) and was given as input to PhyloTreePruner (v. 1.0 [57]). Following the workflow of Bazinet *et al*. [58], orthologous groups that showed evidence of out-paralogues for any taxa were discarded; for those with in-paralogues, multiple sequences were combined into a single consensus sequence for each taxon. This process left 839 orthologous groups eligible for inclusion in data matrices. Individual orthologous group alignments were then concatenated (*nt123_unfiltered* matrix). Positions not represented by sequence data in at least four taxa were then removed (*nt123* matrix), which resulted in more compact data matrices. To address potential issues in regards to missing data, three additional matrices were generated by increasing the required representation for a position to remain within the data matrices: *nt123_min80percentcomplete* (position must have been represented in at least 16 taxa), *nt123_min90percentcomplete* (position must have been represented in at least 18 taxa) and *nt123_100percent complete*(position must have been represented in all taxa).

The *nt123* nucleotide matrix was then subjected to degen1 encoding (v. 1.4 [59]), which we refer to as our *degen* matrix. ‘Degen’ uses degeneration coding to eliminate all synonymous differences among species from the dataset, resulting in phylogeny inference based only on non-synonymous nucleotide change. This procedure was shown in a previous study [60] to generally improve recovery of deep nodes.

### 2.6 Phylogenetic analyses

To conduct the phylogenetic analyses, we used Genetic Algorithm for Rapid Likelihood Inference (GARLI, v. 2.1 [56]) through the GARLI Web service hosted at molecularevolution.org [61]. We used a general time reversible nucleotide model [62] with a proportion of invariant sites and among site rate heterogeneity modelled with a discrete gamma distribution (GTR+I+G) together with GARLI default settings, including stepwise-addition starting trees. We first analysed the *nt123_unfiltered*data matrix, partitioned by codon position (*nt123_partitioned*; electronic supplementary material, figure S1), followed by the unpartitioned *nt123* and *degen* data matrices (*nt123* and *degen* analyses, respectively; electronic supplementary material, figures S2 and S3). We then analysed the partitioned *nt123_unfiltered* data matrix including only the first and second codon positions (*nt12_partitioned*; electronic supplementary material, figure S4) and the three more complete matrices *nt123_min80percentcomplete* (*nt123_min80*; electronic supplementary material figure S5), *nt123_min90percentcomplete* (*nt123_min90*; electronic supplementary material figure S6) and *nt123_100percentcomplete* (*nt123_100percent*; electronic supplementary material, figure S7). For each analysis, we ran 10 best tree searches and 1000 BS replicates. Post-processing of the phylogenetic inference results was performed by the GARLI Web service at molecularevolution.org using DendroPy [63] and the R system for statistical computing [64], which included the construction of a BS consensus tree.

## 3. Results

### 3.1 Read quality statistics

The raw number of 101 bp reads for each newly sequenced transcriptome ranged from 44 805 574 to 65 504 176 (mean: approx. 55 million reads; electronic supplementary material, table S1). Read processing (filtering and trimming) removed 1.40–9.14% of reads per sample (mean: 3.96%); thus, the number of reads provided as input to assembly ranged from 43 047 096 to 63 155 516 (mean: approx. 52 million).

### 3.2 Assembly and data matrix properties

The number of transcript fragments per sample ranged from 56 091 to 242 632 (mean: 108 957; electronic supplementary material, table S2). N50 ranged from 408 to 921 bases (mean: 714). HaMStR results for the 839 orthologous groups used in our analyses are presented in table 2 (HaMStR results for the complete set of orthologous groups are presented in the electronic supplementary material, table S3). The number of sequences from each assembly that matched the HaMStR database ranged from 662 to 1765 (mean: 1172). However, the number of matches to unique orthologous groups ranged from 599 to 1126 (mean: 916). The mean length of matching sequences was 249 amino acids. When concatenated and filtered, the final data matrices contained 9354–1 702 782 nucleotide positions from 839 orthologous groups and were 23.1–100% complete (electronic supplementary material, table S4).

**Table 2.** HaMStR statistics for the subset of orthologous groups passing our paralogy filter, given for each taxon.

species	sequences matching orthologous groups	unique orthologous groups represented	mean length (in amino acids)
*Bathydoris clavigera*	782	621	215
*Doris kerguelenensis*	622	530	190
*Fiona pinnata*	723	623	232
*Pleurobranchaea californica*	918	698	217
*Austraeolis stearnsi*	607	578	238
*Berghia stephanieae*	629	587	228
*Catriona columbiana*	540	513	224
*Cuthona albocrusta*	630	576	239
*Dendronotus venustus*	763	674	263
*Dirona picta*	589	558	227
*Dondice occidentalis*	621	578	227
*Doto lancei*	470	446	205
*Favorinus auritulus*	673	626	255
*Flabellina iodinea*	660	613	264
*Hermissenda crassicornis*	619	593	226
*Janolus barbarensis*	356	351	174
*Melibe leonina*	627	591	229
*Palisa papillata*	615	581	226
*Tritonia festiva*	469	453	168
*Tritoniopsis frydis*	407	388	210

### 3.3 Phylogenetic results

**Figure 3.**
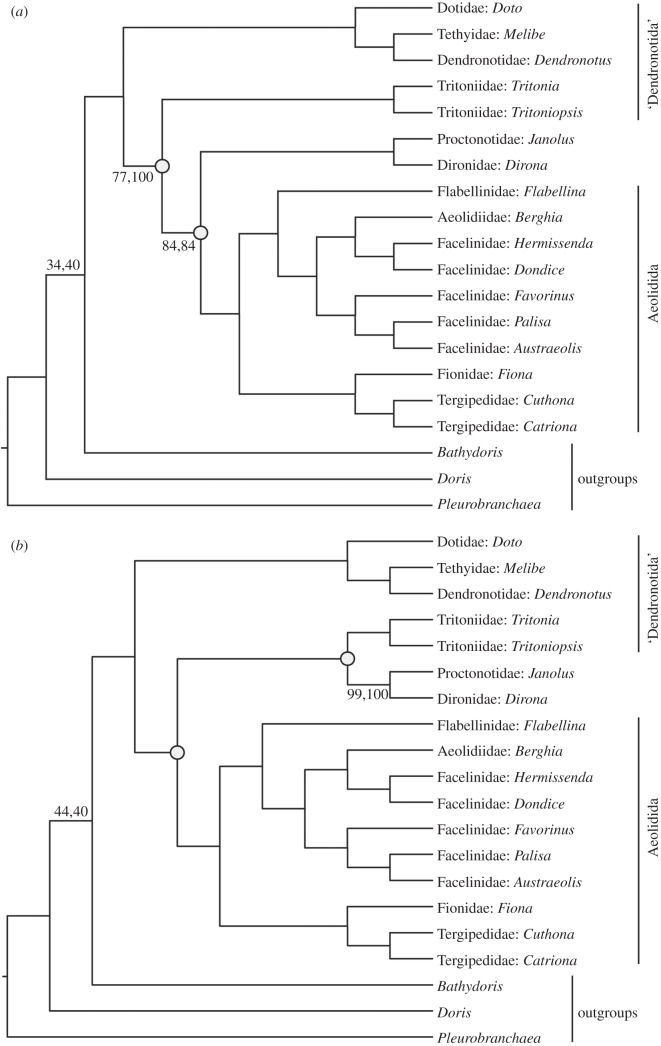
(*a*) The maximum-likelihood tree from the *degen* (first BS value) and *nt12_partitioned* (second BS value) analyses; (*b*) the maximum-likelihood tree from the *nt123* (first BS value) and *nt123_partitioned* (second BS value) analyses. All unlabelled nodes have 100% BS support in both analyses; open circles indicate an inconsistent branching pattern between the two trees.

Our analyses supported Cladobranchia as a monophyletic group with a BS value of 100% (figure 3). This result remains consistent across topologies derived from all seven analyses.

Aeolidida is also monophyletic (BS=100%) across all topologies, containing *Flabellina* (Flabellinidae), *Berghia* (Aeolidiidae), *Hermissenda* (Facelinidae), *Dondice* (Facelinidae), *Favorinus* (Facelinidae), *Palisa* (Facelinidae), *Austraeolis* (Facelinidae), *Fiona* (Fionidae), *Cuthona* (Tergipedidae) and *Catriona* (Tergipedidae). Facelinidae is paraphyletic due to the inclusion of *Berghia* in a clade with the members of this family. This clade is sister to Flabellinidae (BS=100%). In the sister group, Tergipedidae is monophyletic (BS=100%) and is the sister taxon to Fionidae (BS=100%).

Dendronotida is paraphyletic in all topologies. The *degen* and *nt12_partitioned* analyses (figure 3*a*) supported three clades within Dendronotida, and the *nt123 and nt123_partitioned* analyses (figure 3*b*) supported two clades. The clade found in both topologies (BS=100%) contained *Doto* (Dotidae), *Melibe* (Tethyidae) and *Dendronotus* (Dendronotidae) and placed Tethyidae and Dendronotidae as sister groups (BS=100%). The topology returned by the *nt123* analyses included a clade sister to Aeolidida (BS=100% in *nt123* analyses) that contained *Tritonia* (Tritoniidae), *Tritoniopsis* (Tritoniidae), *Janolus* (Proctonotidae) and *Dirona* (Dironidae), whereas the *degen* and *nt12_partitioned* analyses both supported a clade containing Proctonotidae and Dironidae (BS=100%) as sister to Aeolidida (BS=84%, *degen*; 86%, *nt12_partitioned*), and the Tritoniidae clade (BS=100%) as sister to the *Aeolidida*+*Proctonotidae*+*Dironidae* assemblage (BS=77%, 100%).

## 4. Discussion

In this section, we first review the characteristics of our data across our bioinformatics pipeline and the construction of our data matrix. We then address the efficacy of transcriptome data for inferring phylogenetic relationships within Cladobranchia and examine potential methodological concerns. Finally, we discuss the novel results produced by our analyses and compare them to previous studies.

### 4.1 Bioinformatics pipeline and data matrix construction

The number of reads from newly sequenced transcriptomes was higher than from transcriptomes downloaded from the SRA (electronic supplementary material, table S1). This is possibly due to a greater depth of sequencing in our pipeline compared to the original paper that published those transcriptomes [65]. Alternatively, the data retrieved from the SRA had already been filtered, with many reads removed prior to download. Though the average number of transcript fragments from the Trinity assembly was lower in newly generated datasets, the average length of the fragments was higher (electronic supplementary material, table S2). Importantly, the number of sequences that matched to unique orthologous groups, and the average length of these sequences, was sufficient for all taxa (electronic supplementary material, table S3), indicating that sequencing depth may not be a limiting factor. After we removed sites that were represented in fewer than four taxa, overall matrix completeness was slightly higher than in the previous five-gene, 296-taxon analysis [12]. Most importantly, the number of loci used in this analysis was orders of magnitude higher than in the two previous phylogenetic studies on the evolutionary history of Cladobranchia [11,12].

### 4.2 Use of phylotranscriptomics to understand the evolution of Cladobranchia

Phylogenetic inference of Cladobranchia has been a difficult part of the larger problem of understanding the evolutionary history of Nudibranchia [11,12,16,22]. However, the high BS values among the ingroup taxa in our analyses suggest that RNA-Seq will be useful in generating a well-supported hypothesis of the phylogenetic relationships among genera in Cladobranchia, thereby providing a basis for establishing a classification at infraorder, superfamily and family levels that reflects evolutionary history. Our tree topologies are almost fully resolved by our data, and the nodes that are recovered consistently across all trees all have 100% BS support. Thus, a phylotranscriptomic approach for understanding the phylogeny of Cladobranchia seems to be extremely effective in providing evidence to support relationships that were previously uncertain.

### 4.3 Bootstrap support levels

Though our methodology seems to have produced good results, the possibility exists that some of our relatively sparse data matrices (with at most 46.8% completeness) may have misled our likelihood analyses [66]. To address this, we ran analyses with more complete matrices (87.9–100%). In these results, the BS support values are quite high across the phylogenies, even with a matrix length of 48 426 nucleotides (93.6% complete), although the values decrease considerably in the 100% complete matrix analysis. The overall topology of the phylogenies from these analyses was also consistent with the other analyses, with Dendronotida supported as paraphyletic, and Aeolidida and Cladobranchia supported as monophyletic. In addition, some research suggests that missing data may not be as much of a problem as some have suspected. In Cho *et al*. [67], a data matrix with 45% intentionally missing data yielded no signs of the contradictory groupings that missing data might produce. This result is consistent with those of four other studies from across a broad taxonomic range, including frogs [68], angiosperms [69], an entire phylum of eukaryotes [70] and strains of the HIV virus [71].

In addition to concerns regarding missing data, multiple studies have been published that suggest that BS values in phylogenomic analyses may be inflated, primarily due to incongruent gene topologies [72,73]. However, others have suggested that these issues are not as problematic as they may seem. In particular, Simmons & Norton [74] specifically state that BS methods do not seem to have an elevated false-positive rate, and Betancur *et al.* [75] found that the incongruence in the data of Salichos & Rokas [72] is probably the result of sampling error, and thus not likely to be responsible for inflated BS values. In the case of our data and analyses, it is important to note that the divergences within Nudibranchia are much more recent than those addressed by Salichos & Rokas [72], and the number of genes in our analyses surpasses those in Dell'Ampio *et al.* [73].

### 4.4 The phylogeny of Cladobranchia

These analyses have resolved several questions regarding the evolutionary relationships within Cladobranchia. First and foremost, the monophyly of Cladobranchia is reinforced with 100% BS support. Though monophyly was indicated in previous morphological [18] and molecular [12,16] analyses, there have also been studies suggesting paraphyly [11] when the genus *Melibe* was included.

Of the three traditional taxonomic divisions within this group, members of Dendronotida (*Melibe*, *Dendronotus*, *Tritonia* and *Tritoniopsis*) and Aeolidida (*Flabellina*, *Berghia*, *Hermissenda*, *Dondice*, *Favorinus*, *Palisa*, *Austraeolis*, *Fiona*, *Cuthona*and *Catriona*) are included in our analyses, as well as three taxa (*Doto*, *Dirona* and *Janolus*) that were recently classified as unassigned to any of the three groups [11,16,20]. Both *Janolus* and *Dirona* were originally considered to be within Arminida, and *Doto* was once placed under Dendronotida before newer molecular analyses rejected those classifications [15,16,18]. In our analyses, Dendronotida is not supported as monophyletic, which is consistent with previous morphological [18] and molecular [11,12,16,20] phylogenetic hypotheses. Our results indicate a serious need for complete taxonomic revision of the taxa within this group. The monophyly of Aeolidida has also been uncertain. Molecular analyses of Nudibranchia have supported Aeolidida as both paraphyletic and monophyletic, depending on the genes used for the analysis [16]. Other molecular studies on the evolution of Cladobranchia have suggested that Aeolidida is monophyletic [11,20], with very low support, although another did not support Aeolidida as monophyletic [12]. Our analyses strongly support the hypothesis in Pola & Gosliner [11], with Aeolidida being monophyletic with extremely high BS support.

Given our taxon sampling, the earliest diverging lineage within Cladobranchia is a clade containing Dotidae (*Doto*), Tethyidae (*Melibe*) and Dendronotidae (*Dendronotus*), which is a result novel to this study. An exciting result is the inclusion of *Melibe* well within Cladobranchia. This particular genus of filter feeders, which captures crustaceans using a dome-like oral hood fringed by sensory tentacles [76], was excluded from Cladobranchia in a previous study [11]. In that study, the authors attributed this result to a deletion in a section of the COI fragment used in the phylogenetic analyses. Further examination included *Melibe* within Cladobranchia [12], but the *Melibe* clade was at the end of a long branch, and might therefore have caused issues in phylogenetic inference [77].

Elsewhere among the dendronotid taxa, the family Tritoniidae (*Tritonia* and *Tritoniopsis*) is supported as monophyletic, as is a group composed of Proctonotidae (*Janolus*) and Dironidae (*Dirona*). In previous molecular analyses, Tritoniidae has been revealed as both paraphyletic [12] and monophyletic [11], though the monophyly results were poorly supported, with a posterior probability below 0.6. In our analyses, the exact positions of these two clades are uncertain. In one of our analyses (*nt123_unfiltered*), these two clades are sister taxa (figure 1*b*), whereas other datasets (*nt123* and *degen*) suggest that the clade consisting of Proctonidae and Dironidae is the sister taxon of Aeolidida. Neither of these cases were supported in any previous analyses, but the tree presented in figure 3*b* clearly provides stronger support for the two clades as sister taxa, and these analyses included both synonymous and non-synonymous substitutions.

Within Aeolidida, the families Tergipedidae (*Cuthona* and *Catriona*) and Fionidae (*Fiona*) are sister taxa, supporting a previous hypothesis [22]. The study of Carmona *et al*. [11] weakly supported these taxa as an early diverging lineage within Aeolidida. Here, our phylogenomic data strongly favour this clade as the sister group of the remaining aeolid taxa in our analysis. An especially interesting result within Aeolidida is the paraphyly of Facelinidae (*Hermissenda*, *Dondice*, *Favorinus*, *Palisa* and *Austraeolis*), which forms a clade with Aeolidiidae (*Berghia*) that is sister to Flabellinidae (*Flabellina*). The paraphyly of Facelinidae with respect to Aeolidiidae is consistent with the results of Carmona *et al*. [22] and Mahguib & Valdés [20]. Our data strongly support the hypothesis that Aeolidiidae is derived from within Facelinidae and that the closest relative to Aeolidiidae, among the facelinid taxa we have been able to include, is a clade consisting of *Hermissenda* and *Dondice*, two genera that were not included in the Carmona *et al*. study [18]. Our lone representative of Flabellinidae was revealed as the sister group of Facelinidae plus Aeolidiidae. Similarly, Carmona *et al*. [18] found flabellinids in a clade sister to a clade consisting of Facelinidae, Babakinidae and Aeolidiidae, but their study revealed Flabellinidae to be polyphyletic, being interspersed with taxa from the family Piseinotecidae, which has not yet been sampled for phylogenomic data. Hence, broader taxon sampling will be necessary to better understand the specificity of these evolutionary relationships within Aeolidida.

Though this study supports some previous hypotheses and provides new support for others, more work remains to be done. It is critical for future studies to increase taxon sampling. These analyses represent only 10 of the 32 families and 17 of the over 100 genera from Cladobranchia that are accepted in the World Register of Marine Species [78]. It will be especially important to include taxa from Arminida, which would allow for a more complete evaluation of the placement of families with uncertain affinities. Additionally, a representative from Doridoxidae would allow for evaluation of the placement of *Doridoxa* within Cladobranchia [20], for which existing molecular data yield inconsistent results [21]. Overall, an increase in the number of genera and families represented in these analyses will allow for a more complete hypothesis regarding the evolutionary history of Cladobranchia, which will in turn allow for assessments of character evolution through detailed comparative analysis.

## Supplementary Material

Figure S1.

## Supplementary Material

Figure S2.

## Supplementary Material

Figure S3.

## Supplementary Material

Figure S4.

## Supplementary Material

Figure S5.

## Supplementary Material

Figure S6.

## Supplementary Material

Figure S7.

## Supplementary Material

Table S1.

## Supplementary Material

Table S2.

## Supplementary Material

Table S3.

## Supplementary Material

Table S4.

## Supplementary Material

GASTRO50

## Supplementary Material

nt123_100percentcomplete.txt

## Supplementary Material

nt123_min80percentcomplete.txt

## Supplementary Material

nt123_min90percentcomplete.txt

## Supplementary Material

nt123_unfiltered_data_matrix.nex

## Supplementary Material

degen_data_matrix.txt

## Supplementary Material

nt123_data_matrix.txt

## Data accessibility

Transcriptomes can be accessed at the SRA at NCBI: SRA accession nos. SRR1950939–SRR1950954. Aligned data matrices and the GASTRO50 custom substitution matrix are included as the electronic supplementary material to this paper.
